# Delivery of Sumac (*Rhus coriaria* L.) Extract in a Chewing Gum System and Its Functional, Textural, and Sensory Characterization

**DOI:** 10.1002/fsn3.70063

**Published:** 2025-03-05

**Authors:** Alireza Ostadrahimi, Shiva Ezzati, Mir Babak Bahadori, Roghayeh Molani‐Gol, Vahideh Ebrahimzadeh Attari, Ehsan Moghaddas Kia

**Affiliations:** ^1^ Nutrition Research Center Tabriz University of Medical Sciences Tabriz Iran; ^2^ Department of Food Science and Technology, Faculty of Agriculture University of Tabriz Tabriz Iran; ^3^ Medicinal Plants Research Center Maragheh University of Medical Sciences Margheh Iran; ^4^ Student Research Committee Tabriz University of Medical Sciences Tabriz Iran; ^5^ Department of Nutrition and Food Sciences Maragheh University of Medical Sciences Margheh Iran; ^6^ Department of Biochemistry and Nutrition, Faculty of Nutrition and Food Sciences Tabriz University of Medical Sciences Tabriz Iran

**Keywords:** antioxidant activity, chewing gum, *Rhus coriaria* L., sensory properties, sumac, texture profile analysis

## Abstract

Sumac ( *Rhus coriaria* L.) is a rich source of polyphenols and anthocyanins with potential antimicrobial, anti‐inflammatory, and antioxidant characteristics. Regarding the different health‐promoting effects of *Rhus coriaria* L., the present study aimed to formulate and investigate multiple attributes of a sugar‐free medicated chewing gum containing sumac extract. Accordingly, we incorporated different concentrations of a freeze‐dried ethanolic extract of sumac (0%–20%) into a sugar‐free chewing gum to develop a functional food. Then, the produced samples were characterized according to their textural, color, sensory, and functional properties. The results indicated that the antioxidant and phenolic content of chewing gums significantly increased by increasing sumac extract amounts (*p* < 0.05). All samples had favorable sensory scores, which show the suitability of sumac usage. Texture parameters, like chewiness and firmness of samples, were influenced by the sumac addition, while the Chroma index was intensified in a higher amount of sumac (*p* < 0.05). In conclusion, applying sumac bioactive compounds may be a good solution for food industries to deliver decent plant‐based pigments and phytocompounds in sugar‐free products that can be used as functional foods for different preventive/therapeutic purposes.

## Graphical abstract

Sumac is a rich source of polyphenols and anthocyanins with potential antimicrobial, anti‐inflammatory, and antioxidant characteristics. According to our findings, the antioxidant and phenolic content of sumac chewing gum significantly increased in a dose‐dependent manner by increasing the sumac extract amount, and all samples had favorable sensory scores, which show the suitability of sumac usage.

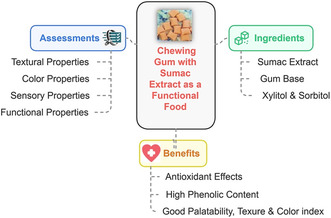

## 1 Introduction

Phytochemicals are bioactive compounds found naturally in plant foods and contribute to their flavor and color. They may also be added to food products through fortification. Phytochemicals have recently been noticed because of various potential health benefits, including antioxidant, anti‐inflammatory, antimicrobial, antiaging, anticancer effects, and improved metabolic functions (Yang and Ling 2024). Foods and food products with biologically active ingredients that have health benefits over their nutrient components are known as functional foods (Oladimeji and Adebo 2023).

While there are various methods, such as pills, tablets, and fortified foods, to provide these beneficial compounds, there is a requirement for a straightforward and affordable process that ensures easy accessibility and use, the stability of these active agents during extended storage, improved absorption, along with satisfactory taste and texture in the final product (Yang and Ling 2024; Guaadaoui et al. 2014).

Chewing gum has the potential to serve as a distinctive carrier for bioactive compounds, offering the possibility of preventing and alleviating various diseases while also satisfying consumers (Hosseini et al. 2024; Bhoi and Pimpodkar 2015; Palabiyik et al. 2018). Research showed that chewing gum is associated with some health benefits like oral hygiene, inhibition of dental caries, treatment of xerostomia (Leveille et al. 2008), improvement in cognitive functions (Curro 2008; Onyper et al. 2011), and decreasing stress and appetite (Bobillo et al. 2018; Melanson and Kresge 2017; Oladimeji and Adebo 2023).

Chewing gum consists of an elastic and stretchable material made up of two phases, each containing different compounds depending on the type of chewing gum. Typically, it is produced by mixing an insoluble component called gum base with soluble flavorings and sweeteners. There are four main categories of chewing gums: sugar, sugar‐free, coated, and medicinal. Sugar‐free gums are produced by replacing glucose syrup and sugar with high‐intensity sweeteners and sugar alcohols in traditional formulations, which are preferred by those looking to limit their sugar intake for reasons like oral health, calorie reduction, diabetic concerns, and others (Hosseini et al. 2024; Konar et al. 2016).

*Rhus coriaria* L., also recognized as sumac, is classified within the Anacardiaceae family and grows in subtropical and temperate regions worldwide. The Dried sumac fruits, with a deep red color and sour flavor, are commonly used as a spice in the Middle East, especially in Turkey and Iran (Alsamri et al. 2021; Karadaş et al. 2020; Langroodi et al. 2018). It has over 180 bioactive components, including anthocyanins, tannins, gallic acid, fiber, and essential oils (Alsamri et al. 2021; Rayne and Mazza 2007). The sour flavor of sumac is primarily attributed to the presence of citric acid and malic acid (Fereidoonfar et al. 2019; Karadaş et al. 2020).

The antioxidant and antibacterial properties of sumac extract are mainly due to its phenolic components, such as tannins, gallic acid, and various flavonoids (Alsamri et al. 2021; Langroodi et al. 2018). Various beneficial pharmacological activities of sumac have been reported, including antimicrobial, anti‐inflammatory, antioxidant, anticancer, hypolipidemic, hypoglycemic, and weight‐lowering effects (Alsamri et al. 2021; Batiha et al. 2022; Elagbar et al. 2020; Khoshkharam et al. 2022; Paradkar et al. 2016). The use of sumac is rapidly increasing not only for culinary applications and as a medicinal herb but also as a food fortifier, food coloring, and food preservative agent in the food industry (Kizil and Turk 2010; Pakseresht et al. 2023; Roberts and Wright 2012; Sakhr and El Khatib 2020).

Generally, sumac has been recently noticed for different dietary investigations because of its palatability and potent antioxidant activity. To our knowledge, there is no study on a medicated chewing gum containing sumac. Thus, the present study aimed to incorporate sumac extract in a sugar‐free chewing gum system along with its physicochemical, sensory, and textural evaluations.

## 2 Materials and Methods

### 2.1 Materials of Chewing Gum

The dried whole fruit of sumac ( *Rhus coriaria* L., Iranian red sumac) was purchased from a local market in Kharvana, Iran. The gum base was prepared from Shoko Ziba Food Ind. (Tabriz, Iran). Pure xylitol, sorbitol (Spectrum Chemical Mfg. Corp.), and glycerol (Mojallali lab Ind.) were supplied by a laboratory equipment store in Tabriz, Iran.

### 2.2 Preparation of Sumac Extract

Initially, the dried fruit of red sumac was wholly ground. An ethanolic extract of the powdered sumac was prepared through the Maceration method 2 kg powder in 10 L of absolute ethanol (99.6% Sepahan Bio‐Product Com.) at 25°C with stirring. The extraction procedure was repeated three times. The resulting solutions were combined and filtered using filter paper to separate solid particles. Subsequently, the solvent was removed by the rotary evaporator (Heidolph, Germany) under vacuum at 40°C. Then, the obtained extract was lyophilized using a laboratory freeze‐dryer (B. Braun Biotech International, Germany) to eliminate residual solvent and enhance the extract's shelf life. Finally, the solvent‐free ethanolic extract with a viscous and dark red appearance was stored at 4°C without light until the chewing gum formulation.

### 2.3 Chewing Gum Preparation

**FIGURE 1:**
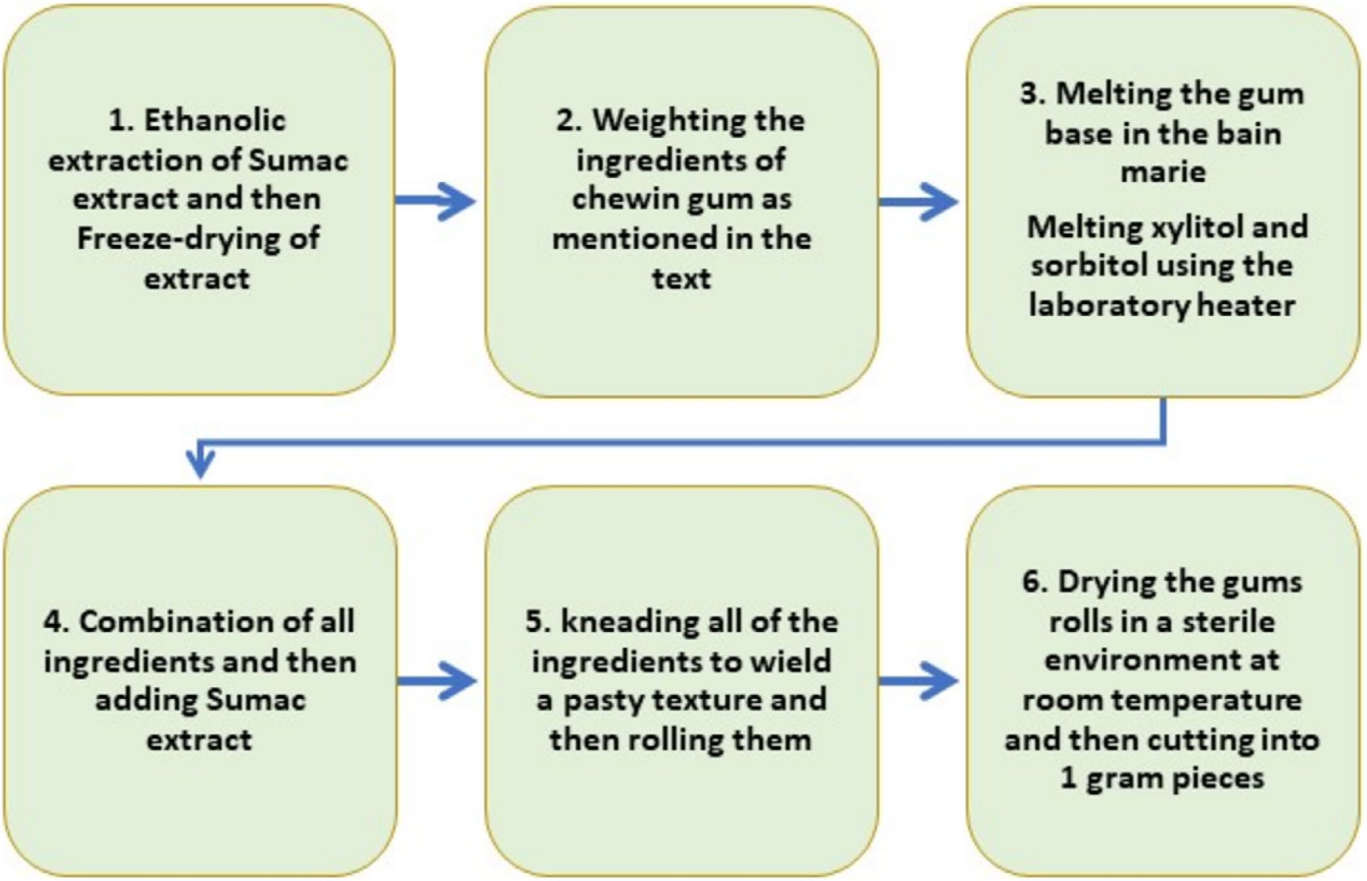
Production processes of Sumac chewing gum.

**FIGURE 2:**
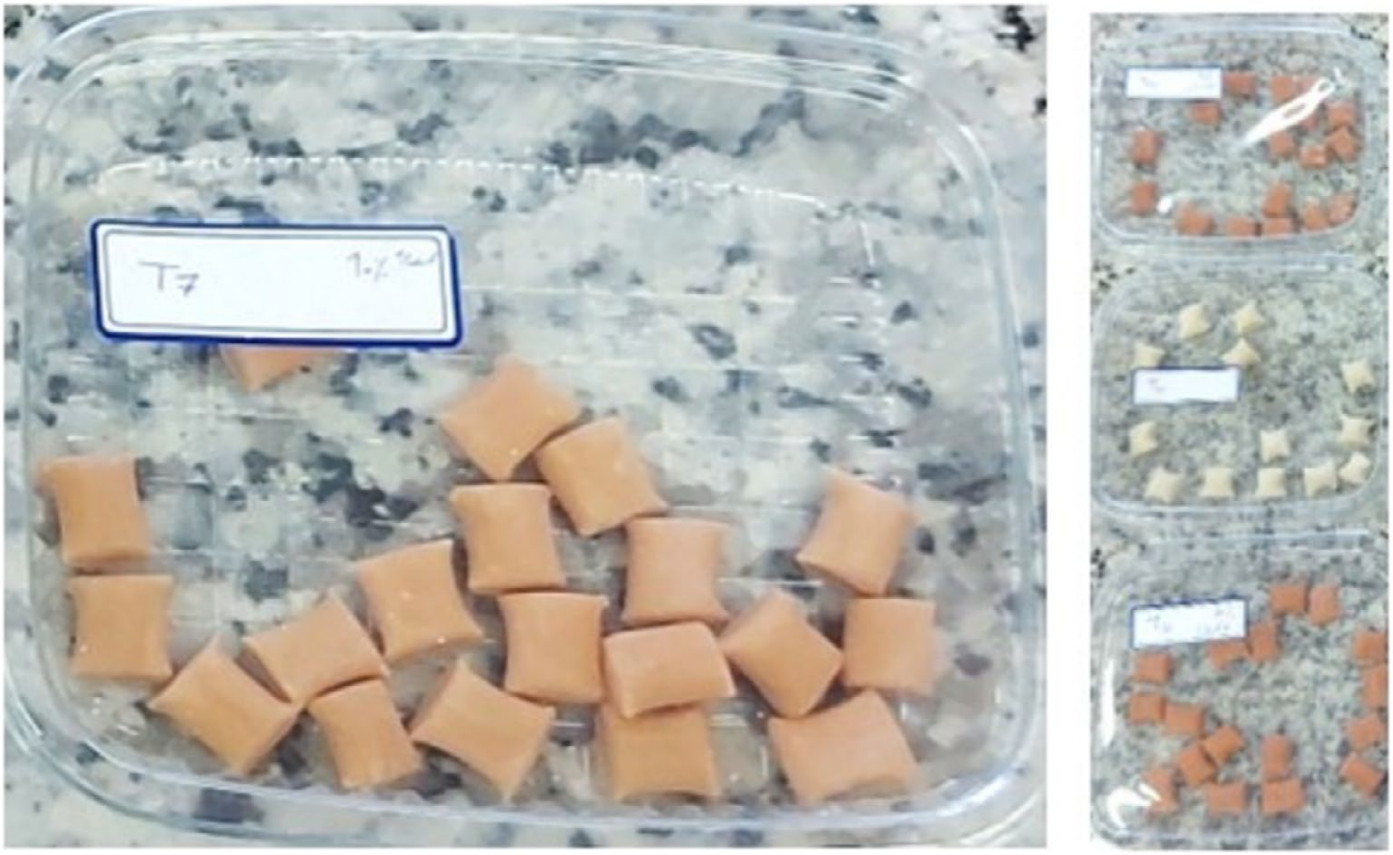
Actual pictures of the samples.

Figure 1 shows the steps of gum production in detail. Different formulations of chewing gum were prepared based on Aboutalebi et al. (2019) protocol with some modifications. According to the study protocol, chewing gums should be prepared in four proportions of sumac extract (5%–20%) to compare samples during final biochemical, textural, and sensory analyses. In this regard, the amounts of gum base, xylitol, and glycerin were constant in all samples, and only the amounts of sorbitol and sumac extract were changed in four samples. So, the final formulation of chewing gums contains 40% of weight (w/w) gum base, 34% w/w xylitol, 3%–18% w/w sorbitol, 3% glycerol, and 5%–20% of the lyophilized sumac extract (5% [sample A], 10% [sample B], 15% [sample C], 20% [sample D]). Moreover, chewing gums were produced with and without the extract (as the control samples for each one). Figure 2 represents actual pictures of the products.

### 2.4 Determination of Total Phenolic Content

The overall phenolic content was measured using the Folin–Ciocalteu reagent with gallic acid as the standard, following a previously outlined procedure with some adjustments (Palabiyik et al. 2018). Briefly, 5 mL of water, 1–3 mL of the sample, and 0.5 mL of Folin–Ciocalteu reagent were incubated for 5–8 min at room temperature. After adding 1.5 mL of 20% w/v sodium carbonate to reach a total volume of 10 mL, the solution was mixed and incubated for 2 h before being filtered through a 0.45 μm polytetrafluoroethylene filter. The absorbance at 750 nm was then measured using a Cecil (CE 3000) UV–VIS spectrophotometer. The total phenol content was quantified by comparing the absorbance of the samples to that of the gallic acid standard. A calibration curve was prepared using the standard gallic acid in the 5–25 mg/L range, and the results were reported as mg of gallic acid equivalence (GAE) per gram of sample. All tests were done in triplicate. The antioxidant and phenol levels of the samples were examined and compared at the start of production and 1 month after storage of chewing gums in closed containers at room temperature.

### 2.5 The Assessment of Antioxidant Capacity

The antioxidant capacity of samples was determined by DPPH radical (2,2‐diphenyl‐1‐picrylhydrazyl) scavenging activity (Bölük et al. 2021). DPPH is a stable chromogenic radical characterized by intense purple coloration. The DPPH assay is based on the electron donation of antioxidants to neutralize the DPPH radical. The reaction is associated with a color change in DPPH, measured by the spectrophotometer at 517 nm, and the degree of discoloration serves as an indicator of antioxidant efficacy (Shahidi and Zhong 2015). A methanolic solution of DPPH radical at a concentration of 100 μM was employed. The samples were diluted with methanol to create various concentrations. Equal amounts (750 μL) of DPPH solution and sample solutions were combined. After 50 min of sitting at room temperature, the absorbance was measured, where DPPH exhibits the maximum absorbance.

### 2.6 Sensory Analysis

The sensory characteristics of chewing gums were investigated using the 5‐point hedonic method with some modifications (Saberi et al. 2018). A sensory evaluation test was done by 15 panelists, consisting of female and male adults aged 20–40 years. The participants tested chewing gum samples during five separate sessions, with consistent conditions including lighting, containers, water for rinsing, sample coding, and presentation order. The participants chewed the samples for 10 minutes. The evaluation was conducted for different parameters such as appearance, chewiness, aroma durability, hardness, adhesiveness, sweet taste, and overall acceptability.

### 2.7 Texture Profile Analysis (TPA) of Chewing Gums

The texture properties of chewing gums were determined using a texture analyzer (Stable Microsystems, TA. HD Plus) equipped with a 5 kg load cell. Mechanical properties are determined by subjecting a bite‐size of materials to two compression cycles (two‐bites test) in a reciprocating motion that simulates the action of jaws where high compression force is used to imitate teeth chewing. This test produces a forc–displacement response curve, from which various textural parameters, closely correlated with sensory evaluation, can be derived and applied to assess chewing gums (Al Hagbani and Nazzal 2018a). The gum samples were cut into equal sizes (10 × 4 mm). The analysis was carried out using a probe with a diameter of 15 mm and were compressed to 20% of their original height. The crosshead speed was considered to be 2 mm/s. The results were presented as the mean value derived from three replicates.

### 2.8 Color Parameters

The samples were analyzed for color parameters including brightness (L*), redness/greenness (a*), and yellowness/blueness (b*) using a colorimeter (Chroma Meter CR‐400, Konica Minolta, Japan). The L* values quantify the degree of brightness, ranging from 0 to 100. The a* value represents the spectrum from red to green, where a positive value indicates red and a negative value indicates green. The b* value represents the spectrum from yellow to blue, with a positive value indicating yellow and a negative value indicating blue. Based on L*, a*, and b* values, C* (chromaticity) and *H* (hue angle) were also measured (Caliskan and Dirim 2016).

### 2.9 Statistical Analysis

The data were analyzed using SPSS software version 21. Descriptive statistics, including mean and standard deviation, and frequency and percentage, were used for quantitative and qualitative variables, respectively. To examine possible differences between samples, the one‐way ANOVA with Tukey's post hoc test and the Kruskal–Wallis test were utilized for quantitative and qualitative variables, respectively. Moreover, the paired‐sample *t*‐test was used to assess time‐dependent within‐group differences in antioxidant activity and phenolic content. Statistical significance was considered based on a *p* value < 0.05.

## 3 Results and Discussion

### 3.1 Formulation of Chewing Gum Containing Sumac Extract

Chewing gums are a promising candidate for delivering different bioactive compounds (Aslani and Rostami 2015; Bobe et al. 2024; Chandran et al. 2014; Konar et al. 2016). The demand among consumers for food products that are safe and nutritious and possess high organoleptic quality is steadily increasing. To our knowledge, this is the first time that a medicated chewing gum containing sumac extract has been formulated and produced. We also used sugar alcohols, including xylitol and sorbitol, as a healthy substitute for sugar. It was established that sugar‐free chewing gums exert an anticariogenic effect attributed to stimulating saliva during the chewing process and the existence of dietary polyols. Xylitol and sorbitol are the most commonly utilized dietary polyols in sugar‐free chewing gums (Edgar 1998; Mickenautsch et al. 2007). Most oral bacteria cannot metabolize Xylitol and Sorbitol, triggering saliva production (Aluckal and Ankola 2018).

### 3.2 Antioxidative Capacity and Total Phenolic Content

Sumac ( *Rhus coriaria* L.) is a spice and functional food with significant antioxidant activity, attributed to its high content of phenolic compounds (Perrone et al. 2022). The effective investigation of natural antioxidant sources necessitates using reliable methods for assessing the antioxidant activity. Regarding the different advantages of the DPPH assay, including its simplicity, high reproducibility, and suitability for operation at room temperature, it is one of the most commonly employed methods. It offers the first approach for evaluating antioxidant activity (Shahidi and Zhong 2015).

The mean and SD of the antioxidant and phenolic properties of chewing gum samples and the controls are shown in Table 1. In order to characterize the stability of antioxidative parameters and retention of total phenolic content in chewing gum samples, these experiments were repeated after 1 month of storage at room temperature. According to Table 1, the antioxidative capacity of all samples ranged from about 77%–88%, which was significantly higher than controls (*p* < 0.05). As expected, the phenolic content increased significantly with the increase in the extract percentage from 5% to 20% w/w (*p* < 0.05). It is noteworthy that sample D, which contained 20% w/w sumac extract, had the highest antioxidant and phenolic contents. Moreover, the antioxidant activity and phenolic content of all chewing gum samples fortified with sumac extract had a slight but statistically significant decrease during 1 month, ranging from 3% to 17%, which was not substantial and indicates a high retention time.

**TABLE 1:** Comparison of the antioxidant capacity and phenolic contents among chewing gum samples^a^.

Samples		Antioxidant capacity^b^		*p* ^e^	Total phenolic content^c^		*p* ^e^
		1st day	30th day		1st day	30th day	
A	Sumac Gum	78.68 ± 0.22	77.38 ± 0.24	< 0.001	3.21 ± 0.01	3.1 ± 0.01	< 0.001
	Control^d^	45.82 ± 2.55	45.79 ± 0.63	0.976	2.34 ± 0.05	2.21 ± 0.07	0.017
	*p* ^f^	< 0.001	< 0.001		< 0.001	< 0.001	
B	Sumac Gum	81.77 ± 0.241	80.19 ± 0.17	< 0.001	3.34 ± 0.01	3.23 ± 0.06	< 0.001
	Control	37.14 ± 0.30	35.66 ± 5.34	0.580	1.89 ± 0.01	1.88 ± 0.20	0.891
	*p* ^f^	< 0.001	< 0.001		< 0.001	< 0.001	
C	Sumac Gum	84.37 ± 0.12	83.33 ± 0.33	0.001	3.71 ± 0.08	3.13 ± 0.001	< 0.001
	Control	17.96 ± 0.99	16.90 ± 3.32	0.456	1.74 ± 0.01	1.73 ± 0.001	0.917
	*p* ^f^	< 0.001	< 0.001		< 0.001	< 0.001	
D	Sumac Gum	89.34 ± 0.61	88.91 ± 0.04	0.380	4.11 ± 0.07	3.45 ± 0.01	< 0.001
	Control	12.49 ± 0.85	13.71 ± 3.65	0.433	1.91 ± 0.03	1.89 ± 0.001	0.472
	*p* ^f^	< 0.001	< 0.001		< 0.001	< 0.001	
*p* ^g^		< 0.001	< 0.001		< 0.001	< 0.001	

^a^ Data are presented as Mean ± SD.

^b^ Represent as a percent based on DPPH scavenging activity.

^c^ Total phenolic content was expressed as gallic acid equivalents (GAE) in mg per g dry material.

^d^ Chewing gum without Sumac extract.

^e^ Comparisons of each sample before and after 1 month of production are based on a paired‐sample *t*‐test.

^f^ Comparisons of each Sumac Gum sample group with its control sample based on an independent samples *t*‐test.

^g^ Comparison of 4 sample groups based on the ANCOVA test and the post hoc test of Tukey, with the adjustment of control values.

In agreement with our findings, Aydogdu Emir (2023) prepared a guar gum sumac film with intense antioxidant activity. Similarly, Jamous et al. assessed the antioxidant activity of sumac extracts derived from the leaves and fruit epicarps using the free‐radical scavenging capacity (DPPH) and reducing power (RP) assays. The results showed potent antioxidant and pancreatic lipase inhibitory activity with potential applications in treating and preventing obesity and overweight (Jamous et al. 2018). In another study, a sumac‐enriched yoghurt exhibited a significant increase in antioxidant activity and total phenolic content compared to plain yoghurt (Perna et al. 2018). Similar to our study, Aslani and Rostami (2015) formulated a ginger chewing gum with high phenolic content. Moreover, Crowe‐White et al. assessed the effect of chewing two sugar‐free gums formulated by spices (cinnamon or cinnamon + nutmeg) on the antioxidant capacity and phenolic content of saliva. They reported that both gums significantly increased the antioxidant capacity and phenolic content of saliva (Crowe‐White et al. 2023).

The phenolic composition of sumac depends on its genetic makeup, cultivation conditions, harvest timing, and the particular plant parts analyzed (Aydogdu Emir 2023). The primary phenolic acids in sumac are gallic acid, along with caffeic acid and ellagic acid (categorized as hydroxycinnamic acids) (Fereidoonfar et al. 2019; Pakseresht et al. 2023; Zannou et al. 2025). Phenolic compounds are nutraceuticals with health‐promoting properties and therapeutic values (Yang and Ling 2024). Sumac, as both a medicinal plant and functional food, can be considered a potential therapeutic option for chronic diseases, for example, cancer, diabetes, hyperlipidemia, nonalcoholic fatty liver, and Alzheimer's disease, primarily through its anti‐inflammatory and antioxidant action (Alsamri et al. 2021; Batiha et al. 2022; Elagbar et al. 2020; Khoshkharam et al. 2022; Paradkar et al. 2016; Tohma et al. 2019).

### 3.3 Sensory Analysis

Sensory analysis is a powerful tool for evaluating the human appreciation of food products (Basile et al. 2023). Attributes such as appearance, aroma, flavor, and texture are crucial in determining food quality and consumer preference (El Sheikha 2021).

Table 2 represents the comparison of sensory indices, including appearance, color, smell, taste, elasticity, stiffness degree, stickiness, and flavor retention among different sample groups. All samples had favorable high scores in the sensory indices and enough palatability, which shows the proper selection of sumac extract for chewing gums. Our results showed no significant difference between groups except for the color index, in which sample D received the highest score (*p* < 0.05). According to our findings, the flavor retention rate was high in all sumac gums (3–4 scores), possibly due to some essential oils in the sumac extract as the freeze‐dried sumac extract was viscous and slightly viscous. It was reported that the water‐soluble flavor particles of chewing gum are quickly released during mastication, while the flavor particles dissolved in the gum base are slowly released. On the other hand, the hydrophobic flavors show slower and more extended releases than hydrophilic flavors in chewing gums (Hinderink et al. 2019).

**TABLE 2:** Comparison of the sensory characteristics among chewing gum samples^a^.

Properties	Samples				
	A	B	C	D	*p* ^b^
Appearance	4 (2–5)	4 (1–5)	4 (3–5)	4 (2–5)	0.466
Color	4 (1–5)	4 (2–4)	4.5 (2–5)	5 (1–5)	0.007
Smell	3.5 (1–5)	4 (1–4)	4 (1–5)	4 (1–5)	0.417
Taste	4 (1–5)	4 (1–5)	4 (1–5)	3 (1–5)	0.581
Elasticity	4 (1–5)	3.5 (2–5)	4 (2–5)	3 (2–5)	0.73
Stiffness degree	3 (2–5)	3.5 (1–4)	2.5 (1–5)	3 (1–5)	0.234
Stickiness	2 (1–5)	2.5 (1–5)	3 (1–5)	2 (1–5)	0.941
Flavor retention	3 (2–4)	3 (3–4)	4 (3–5)	3.5 (2–5)	0.177

^a^ Data are presented as Median (min‐max).

^b^ Comparison of four sample groups based on the Kruskal–Walliss test.

Different applications of sumac extract have been investigated in the food industry (Osmólska et al. 2022; Sakhr and El Khatib 2019; Wang and Zhu 2018). A recent study examined the sensory effects of adding sumac extract to spinach soup at different doses. Similar to our finding, there were no statistical differences in liking attributes (acceptance, texture, aroma, flavor, and appearance) between soups with different sumac extracts. However, more sumac addition led to a greater perception of color than the control sample (Soleymani Majd et al. 2023). According to our findings, the results of Al‐Marazeeq et al. (2019) showed that adding an aqueous extract of sumac to bread from 0.5% to 5% increased the quality of bread in a dose‐dependent manner. In another study by Dziki et al. (2021) the sensory and antioxidant effects of wheat bread enriched with sumac flour (SF) were assessed. The results showed that adding SF up to 3/100 g increased bread quality.

### 3.4 Texture Profile Analysis

Texture profile analysis (TPA) is a valuable tool used to evaluate the mechanical properties of food products (Al Hagbani and Nazzal 2018b). The results of the TPA test on chewing gums are presented in Table 3. Accordingly, the firmness was significantly decreased by increasing the dose of sumac extract in samples, albeit there was no inverse linear relation. Generally, the gum base absorbs saliva during chewing, which softens the gum. Regarding the viscous properties of freeze‐dried sumac extract, its addition created a softer texture due to its dispersion in chewing gum and more water absorption by sumac particles.

**TABLE 3:** Comparison of the texture profile analysis (TPA) results among chewing gum samples^a^.

Items	Samples				
	A	B	C	D	*p* ^b^
Firmness (Kg)	2.72 ± 0.08	0.84 ± 0.03	0.17 ± 0.01	0.27 ± 0.01	0.001
Cohesiveness	0.56 ± 0.04	0.51 ± 0.02	0.62 ± 0.08	0.55 ± 0.03	0.76
Springiness	0.60 ± 0.01	0.63 ± 0.01	0.97 ± 0.05	0.53 ± 0.02	0.56
Gumminess	1.61 ± 0.001	0.43 ± 0.02	0.12 ± 0.009	0.14 ± 0.02	0.001
Chewiness	0.97 ± 0.01	0.27 ± 0.02	0.12 ± 0.01	0.08 ± 0.003	0.001

^a^ Data are presented as Mean ± SD.

^b^ Comparison of four sample groups based on the one‐way ANOVA and the post hoc test of Tukey.

Cohesiveness, defined as the amount of force needed to disintegrate the sample into smaller particles (Saberi et al. 2018), was not significantly affected by sumac addition. The springiness also had the same pattern among different samples. Previous studies have also shown that springiness is mainly affected by gum base and elastomers (Saberi et al. 2019). Springiness is defined as the extent to which chewing gum regains its original length after the conclusion of the initial compression cycle and before the commencement of the subsequent cycle. The chewiness of samples was also decreased significantly by sumac addition (*p* < 0.05). Chewiness is a measure of how easily food can be chewed, and it is calculated by combining the hardness, springiness, and cohesiveness values (Mohammadi et al. 2018). According to Chen and Opara, food is easier to chew and requires less energy when chewiness is low (Chen and Opara 2013). Our results showed that sumac addition of up to 20% results in easily chewed samples. This phenomenon could be related to sumac water absorption and the softer texture of samples.

In the study by Palabiyik et al. (2018), Kenger gum, derived from the Kenger plant ( *G. tournefortii* ), was utilized to produce biodegradable and edible chewing gums. The study identified hardness and resilience as critical textural parameters for achieving the optimal texture of chewing gum (Palabiyik et al. 2018).

### 3.5 Color Parameters

The visual appeal of color is a significant factor in determining the quality of foods, particularly in products such as sumac, which is commonly used as a coloring agent. Functional food has the potential to offer numerous health advantages to consumers; however, it must also be visually appealing to have successful marketing (Caliskan and Dirim 2016; Grassia et al. 2021). According to Table 4, the L* values varied between 54 and 56–69.31. A low L* value indicates a loss of brightness. Our results showed that L* and a* were enhanced by sumac addition (*p* < 0.05). The results support the idea that the extracts of sumac fruits in polar solvents like distilled water, methanol, and ethanol appear brownish‐red, which becomes darker with increased extraction of polar anthocyanin fragments (Klančnik and Koradin 2024). The addition of sumac extract increases the Hue angles and Chroma. Chroma (C*), the measurable quality of colorfulness, assesses how much a hue differs from a gray color of the same brightness. Higher Chroma values indicate greater color intensity as perceived by humans (Pathare et al. 2013). So, these samples show good saturation, meaning the color is intense.

**TABLE 4:** Comparison of the color parameters among chewing gum samples^a^.

Items	Samples				
	A	B	C	D	*p* ^b^
L*	69.31 ± 0.56	63.31 ± 0.78	58.69 ± 0.69	54.56 ± 1.23	< 0.001
a*	0.67 ± 0.01	3.80 ± 0.10	6.76 ± 0.23	12.05 ± 0.50	< 0.001
b*	25.30 ± 0.71	27.20 ± 0.82	28.03 ± 0.52	27.71 ± 0.80	0.007
H*	25.15 ± 0.65	27.61 ± 1.34	27.95 ± 1.41	30.26 ± 0.95	0.004
C*	0.02 ± 0.001	0.13 ± 0.01	0.024 ± 0.01	0.041 ± 0.01	< 0.001

*Note:* Brightness (L*), redness/greenness (a*), yellowness/blueness (b*), chromaticity (C*), and hue angle (H*).

^a^ Data are presented as Mean ± SD.

^b^ Comparison of four sample groups based on the one‐way ANOVA and the post hoc test of Tukey.

Generally, the bioavailability and controlled release of active ingredients are fundamental issues in the production of medicated chewing gums, and methods like microencapsulation play an essential role in the controlled release of active components as well as the sensory and color analyses. Recently, Grassia et al. studied the feasibility of sumac extract microencapsulation with maltodextrin, cyclodextrin, and gum Arabic through spray drying. The results showed that the highest values of a* and Chroma were recorded with the lowest concentrations of cyclodextrin and maltodextrin (Grassia et al. 2021).

Chranioti et al. (2015) investigated the use of various agents, including maltodextrin, gum Arabic, gum Arabic‐modified starch, modified starch–chitosan, and modified starch–maltodextrin–chitosan, for the microencapsulation of beetroot and saffron coloring extracts through freeze‐drying. Additionally, the powders were incorporated into a chewing gum model system. The chewing gum samples containing extracts encapsulated with maltodextrin–gum Arabic exhibited the highest a* values for beetroot and b* values for saffron, indicating superior protection of the coloring extracts (Chranioti et al. 2015).

## 4 Conclusion

According to our findings, the antioxidant and phenolic content of chewing gums significantly increased in a dose‐dependent manner by increasing the sumac extract amount. The antioxidant activity and phenolic content of all samples had a slight but statistically significant decrease during 1 month, indicating a high retention time. All samples had favorable sensory scores, which show the suitability of sumac usage. The chewiness and firmness of samples were significantly decreased by sumac addition, and the Chroma index was intensified in a higher amount of sumac. Regarding the palatability of sumac and its high antioxidant capacity, applying sumac bioactive compounds may be a good solution for food industries to deliver decent plant‐based pigments and phytocompounds in sugar‐free products that can be used as functional foods for different preventive/therapeutic purposes.

## Author Contributions

A.O. conceptualization and designing the study. S.E. and M.B.B. implementation of research and writing the manuscript. R.M.‐G. writing and editing the manuscript. V.E.A. conceptualization, designing, supervision, and implementation of the study and writing, review, and editing the manuscript. E.M.K. implementation of the study and writing, review, and editing the manuscript.

## Ethics Statement

The protocol of this study was approved by the Ethics Committee of Tabriz University of Medical Sciences (Ethical cod: IR.TBZMED.REC.1400.559).

## Conflicts of Interest

The authors declare no conflicts of interest.

## Data Availability

The data that support the findings of this study are available upon reasonable request from the corresponding author.
